# Induction of apoptosis in B16‐BL6 melanoma cells following exposure to electromagnetic fields modeled after intercellular calcium waves

**DOI:** 10.1002/2211-5463.13760

**Published:** 2024-02-01

**Authors:** Benjamin D. Rain, Adam D. Plourde‐Kelly, Robert M. Lafrenie, Blake T. Dotta

**Affiliations:** ^1^ Behavioural Neuroscience & Biology Programs, School of Natural Science Laurentian University Sudbury ON Canada

**Keywords:** apoptosis, calcium, cancer, electromagnetic fields, intercellular calcium waves, voltage‐gated calcium channel

## Abstract

Exposure to time‐varying electromagnetic fields (EMF) has the capacity to influence biological systems. Our results demonstrate that exposure to time‐varying EMF modeled after the physiological firing frequency of intercellular calcium waves can inhibit proliferation and induce apoptosis in malignant cells. Single exposure of B16‐BL6 cells to a Ca^2+^ EMF for 40 min reduced the number of viable cells by 50.3%. Cell imaging with acridine orange and ethidium bromide dye revealed substantial cellular apoptosis, preapoptotic cells, nuclear fragmentation, and large spacing between cells in the Ca^2+^ EMF condition when compared to the control condition. The ability of Ca^2+^ EMF to influence the proliferation and survival of malignant cells suggests that exposure to specific EMF may function as a potential anticancer therapy.

## Graphical abstract

This study reveals that exposure to time‐varying electromagnetic fields (EMF), mimicking the physiological firing frequency of intercellular calcium waves, can impede cancer cell proliferation and induce apoptosis. There was a remarkable 50.3% reduction in viable cancer cells. These findings support the possibility of utilizing EMFs as a targeted anticancer therapy.

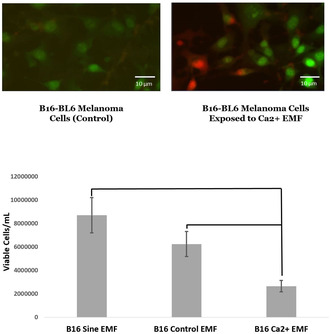

**Abbreviations**
A + A: antibiotic and antimycotic cell culture solution
Act: activator (BAY K8644 L‐type calcium channel activator)
ANOVA: analysis of variance
AO: acridine orange dye
B16‐BL6: B16‐BL6 mouse melanoma cells
BAY K8644: BAY K8644 L‐type calcium channel activator
Ca^2+^
: calcium
Cm: centimeter
CO_2_
: carbon dioxide
EB: ethidium bromide dye
EMF: electromagnetic fields
HEK 293: human embryonic kidney cells
Hz: hertz
IBM SPSS: International Business Machines Statistical Processing Software
ICW: intercellular calcium waves
Ml: milliliter
Mm: millimeter
SEM: standard error of the mean
uT: microtesla
VGCC: voltage‐gated calcium channels
μg: microgram
Ω: Ohm

Biological systems are continuously encompassed by electromagnetic fields (EMF) [1]. These EMFs can be static or dynamic. A dynamic EMF is a propagating field of force that displays temporal variations in intensity and direction. Over the past century, much effort has gone into generating artificial EMFs that emulate those that occur naturally [2, 3]. Modeling the intensities and temporal signatures of the natural EMF environment into an artificial field allows for the generation of indistinguishable patterns from those produced by biological systems [4, 5]. Thus, manufactured EMFs have the potential to elicit naturally occurring focal and global interactions [4]. Complex EMFs, such as those designed to imitate physiologically relevant processes, have demonstrated the ability to modulate the function of the specific process they were modeled after. For example, we have previously demonstrated that the frequency‐modulated ‘Thomas’ patterned EMF alters cell membrane activity [6]. Additionally, rodents exposed to the Thomas field for 180 min·day^−1^ demonstrated impaired memory performance and an increased analgesic response [6, 7, 8].

Calcium signaling is a form of cellular communication essential for the development, conservation, and propagation of many biological systems [9]. This process allows for neighboring cells to communicate with one another and for the entire system to communicate with its environment [10]. Among the most pronounced forms of calcium signaling are intercellular calcium waves (ICW) [11]. Intercellular calcium waves are naturally occurring physiological processes that commence with an increase in Ca^2+^ within a cell that propagates to nearby cells in a wave‐like manner [12]. The process appears to provide the necessary level of communication for a single cell to communicate with its cohort, thereby establishing a multicellular response [12].

Modification of intracellular calcium concentration [Ca^2+^] influences the duration and intensity of the Ca^2+^ wave [11]. Changes in intracellular Ca^2+^ occur via the opening of voltage‐ and ligand‐gated Ca^2+^ channels that allow for a flux of Ca^2+^ across the plasma membrane, or the liberation of Ca^2+^ from internal reserves such as the endoplasmic reticulum (ER) [11]. Alternatively, the Ca^2+^ increase may also occur as a result of Ca^2+^ in the extracellular environment. Voltage‐gated calcium channels (VGCC) are Ca^2+^ permeable ion channels located on the plasma membrane of a cell [13]. There are three principal categories of VGCC: low‐voltage activated (T‐type), high/intermediate voltage‐activated (P/R‐type), and high‐voltage activated (L‐type) [14, 15]. The primary role of VGCCs is to transduce electrical changes from the plasma membrane into an intracellular response [14]. The influx of Ca^2+^ through VGCC is central to the development, proliferation, and apoptosis of a cell; hence, proper functioning of these channels is required for cellular homeostasis [15, 16, 17].

Two different cell lines were employed in this study, B16‐Bl6 and HEK293. The B16‐BL6 cells are derived from a mouse melanoma tumor, while the HEK293 is derived from human embryonic kidney cells. B16‐BL6 cells serve as an adequate model for Ca^2+^ waves due to the high prevalence of T‐type voltage‐gated Ca^2+^ channels, while the HEK293 cells express very low levels of T‐type channels [6, 18, 19]. Activation of T‐type channels occurs following a small membrane depolarization and demonstrates a unique pattern of activation/inactivation present at low voltage (18). Previous biomolecular studies have demonstrated the ability to specific patterned EMFs to influence T‐type calcium channels associated with the inhibition of malignant cell proliferation [4]. Furthermore, T‐type voltage‐gated Ca^2+^ channels have been shown as a mechanism for malignant cell signaling by which intercellular communication occurs as a traveling wave of Ca^2+^ [20]. Because of this previous research, both a malignant (B16BL6) and nonmalignant (HEK293) cell line were chosen.

Due to the aforementioned research, it was of interest to determine whether an EMF modeled after the physiological firing frequency of an ICW could influence Ca^2+^ signaling in the absence of pharmacological or mechanical intervention. It was our hypothesis that an EMF patterned after ICW would decrease the number of viable B16‐BL6 cells and promote apoptosis. We hypothesized, based on previous research, that this effect would occur predominantly in the B16‐BL6 cells, due to the high amount of T‐type voltage‐gated Ca^2+^ channels.

## Materials and methods

### Cell maintenance

B16‐BL6 and HEK293T cells were obtained from the American Type Culture Collection (Manassas, VA, USA) and cultured on 100 mm medium‐adherence culture plates in DMEM/high glucose media (Hyclone; Thermo Fisher, Mississauga, ON, Canada) supplemented with 10% fetal bovine serum and 1% antibiotic and antimycotic (A + A). Cells were subcultured 1 : 5 biweekly and maintained in a culture incubator at 37 °C, 5% CO_2_, and 100% humidity. For experiments, the cells were harvested and approximately 200 000 cells were added to a 60 mm medium‐adherence experimental plate and cultured overnight before treatments.

For some experiments, the cells were treated with 5 μm BAY K8644, a VDCC activator, immediately before exposure to the sham or EMF conditions. Experimental plates were then placed in the incubator at 37 °C, 5% CO_2_ for 48 h.

### EMF exposure

On the day of EMF exposure, experimental plates were gathered from the incubator and treated according to their labeled treatment condition. Control (sham‐treated) plates were placed in a dark Styrofoam box at 22 °C for the duration of the 40‐min exposure time. The box was kept in a separate room more than 10 feet away from the EMF exposure apparatus. Experimental plates (Ca‐EMF) or Sine‐EMF field conditions were placed in the dark, in the center of a field generator at 22 °C. The EMF was applied for 40 min. Following exposure, cells were removed from the field generator and returned to the incubator at 37 °C, 5% CO_2_ for 48 h.

### Field design and application

**Fig. 1:**
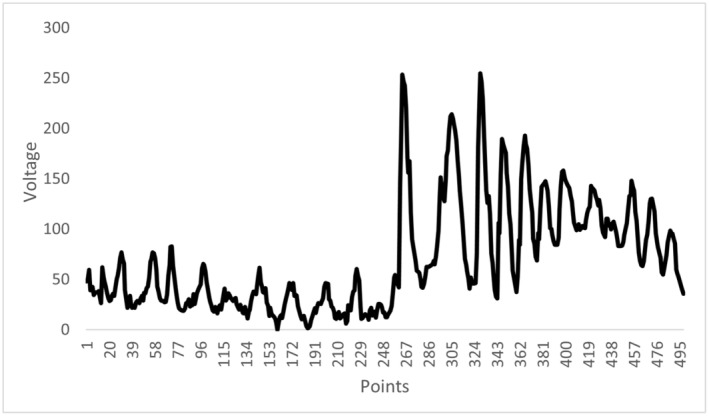
Activation pattern of intra‐ and intercellular calcium waves in murine cells located in the longitudinal layer of the cecum and colon, modeled after Hennig *et al*. [21].

The EMF employed in this study (Fig. 1) was modeled after the physiological activation pattern of calcium waves in murine cells located in the longitudinal layer of the cecum and colon. The pattern of EMF was modeled after a study conducted by Hennig *et al*. [21], in which intra‐ and intercellular calcium wave activity was recorded. From these recordings, we then plotted a graph displaying the relative amplitudes of calcium wave activity. The resulting pattern of this graph was used to design an EMF with identical amplitude, frequency, and temporal characteristics to a naturally occurring calcium wave. The EMF was generated using a digital‐to‐analog device created by Stanley Koren [22]. It is important to note that our EMF design was modeled after the physiological firing pattern of calcium waves and was not designed to be a perfect replication. In this case, we are concerned with modeling the appropriate pattern rather than matching the exact amplitudes. The resulting amplitudes of the Ca^2+^ EMF are proportional to the original pattern, with the maximum intensity emitted by the EMF being 4uT. The pattern completed a full cycle in approximately 5 s. This pattern was generated by a computer, which was connected to an EMF‐generating device, inducing an electric current. The generating device was a 30 Ω Helmholtz coil comprised of a 39 cm × 39 cm box wrapped by 305 m of 30 American Wire Gauge insulated copper wire. All EMFs employed in this study were emitted at an intensity of 1 microtesla which coincides with the intensities of numerous biomolecular pathways. Cell plates were placed in the middle of the generating device and left in the dark for the entire 40‐min duration of the field exposure. The passing of current horizontally through the copper wire generated a corresponding magnetic field of approximately 4 μT in the vertical direction which then passes inside the generating device onto the cell plates.

### Cell counting

Experimental plates were removed from the incubator 48 h following treatment. Cells were harvested and counted [23] using a standard Neubauer ruled 1 mm^2^ hemocytometer and inverted phase‐contrast microscope. The number of viable and nonviable cells present in one 0.25 × 0.20 mm square was counted in duplicate measures, and the number of cells·mL^−1^ was calculated.

### Cell imaging

Experimental plates were removed from the incubator 48 h following treatment. All cells were treated with 5 μg·mL^−1^ acridine orange and 5 μg·mL^−1^ ethidium bromide for 15 min prior to imaging. Images were captured using a Zeiss Axiovert 200 M zoom inverted microscope using the bright field, red, and green channels using the ZEN 3.5 Blue Edition editing software created by Zeiss (North York, ON, Canada).

### Statistical analysis

All data were computed using ibm spss statistical analysis software version 28 (IBM, Markham, ON, Canada). The data presented for the number of viable cells were computed from raw scores tallied using the hemocytometer counting procedure. Additionally, the data displaying percent viability were calculated using a formula obtained from the Sigma‐Aldrich protocol [23]. Levene's test was used to test for homogeneity of variance for both number of viable cells and percent viability. A one‐way ANOVA was conducted, and an independent samples *t*‐test was performed to compare subsets of those data that were normally distributed. In data that violated the homogeneity of variance assumption, a Kruskal–Wallis and Mann–Whitney U independent samples test was used to compare data subsets. No outliers were excluded from the statistical analyses.

## Results

### Reduction in B16‐BL6 viability following exposure to Ca^2+^ EMF

**Fig. 2:**
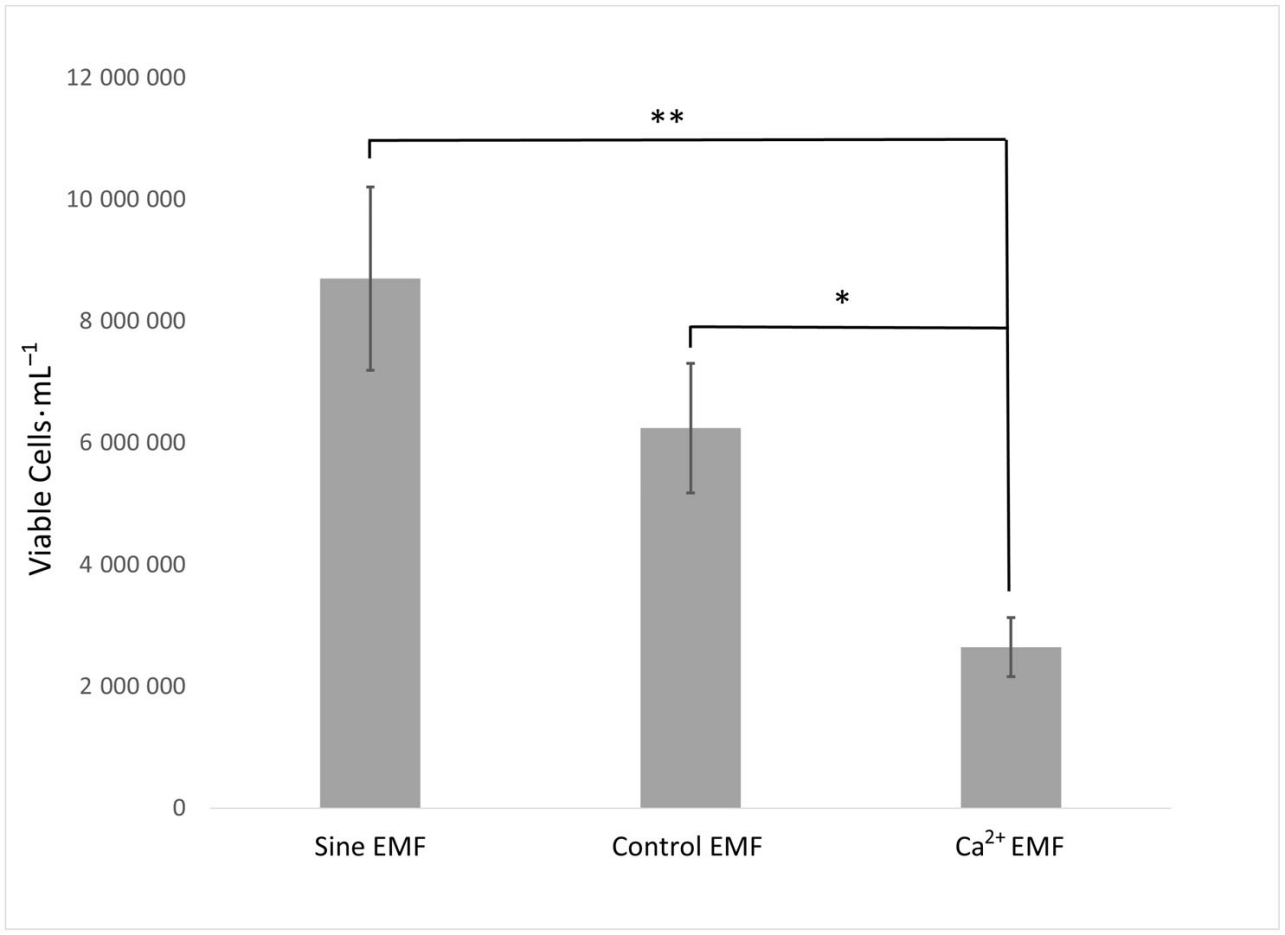
Mean number of viable B16‐BL6 cells by electromagnetic field (EMF) condition, no activator. Data analysis revealed significant differences between the Ca^2+^ EMF condition and No EMF condition [*T*(36) = 2.36, *P = 0.012] and the Ca^2+^ EMF condition and Sine EMF condition [*H*(1) = 10.931, **P < 0.001]. Statistical analysis conducted using *t*‐test (*T*) and Kruskal–Wallis test (*H*). Sine EMF *n* = 8, Control EMF *n* = 16, Ca^2+^ EMF *n* = 10. Error bars represent SEM.

**Fig. 3:**
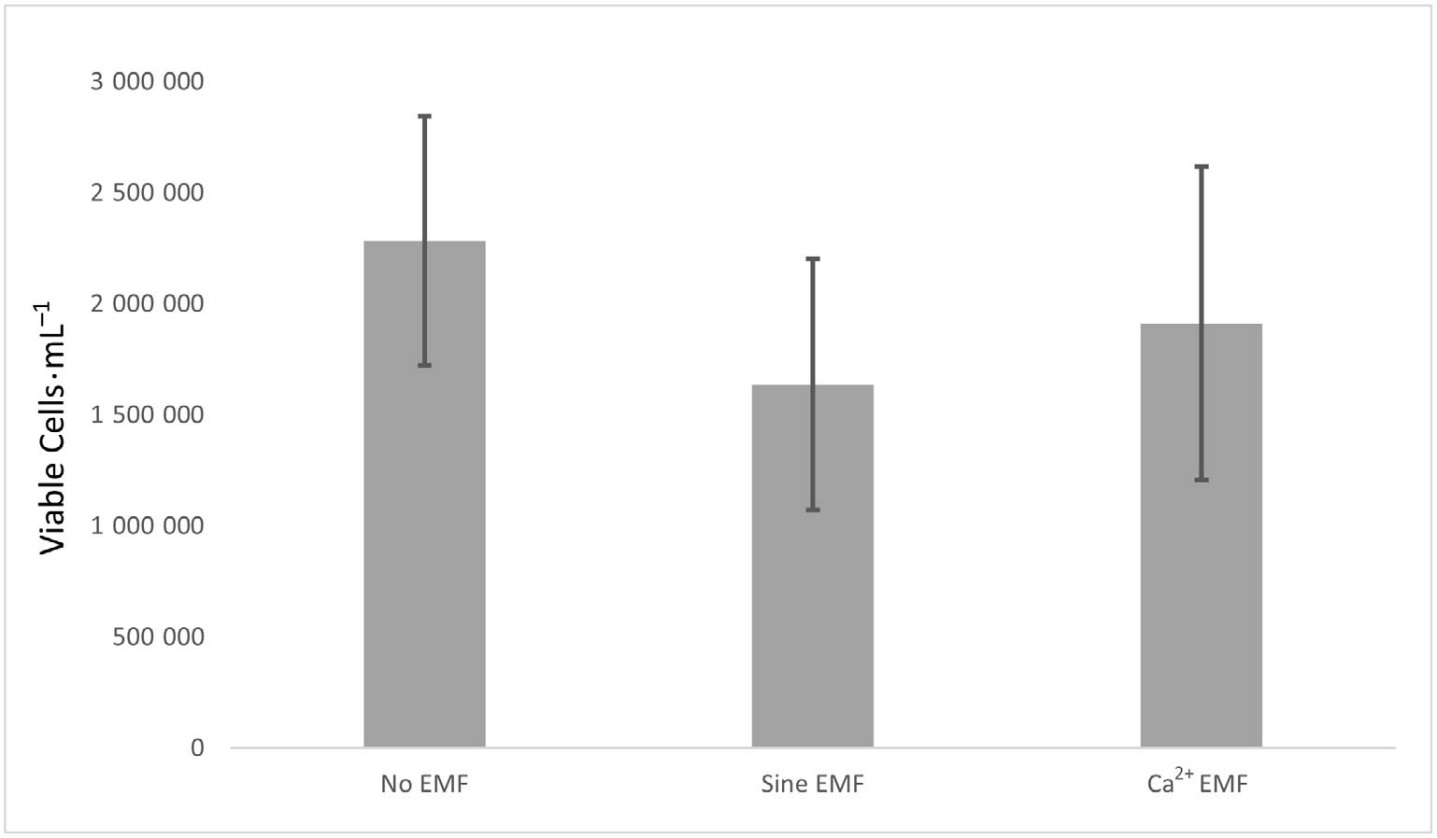
Mean number of viable HEK293T cells per electromagnetic field (EMF) exposure condition. Statistical analysis revealed no significant difference in the number of viable cells between exposure conditions. Statistical analysis conducted using *t*‐test. No EMF *n* = 16, Sine EMF *n* = 10, Ca^2+^ EMF *n* = 12. Error bars represent SEM.

A significant effect was observed between the three treatment conditions (No EMF, Sine EMF, Ca^2+^ EMF), and the number of viable cells counted [*F*(2, 46) = 5.77, *P* = 0.006]. An independent samples *t*‐test revealed that cells subjected to the Ca^2+^ EMF condition had significantly fewer viable cells (50.3%) when compared to the No EMF condition [*T*(36) = 2.36, *P* = 0.012] (Fig. 2). Furthermore, cells subjected to the Ca^2+^ EMF condition displayed significantly fewer viable cells than those in the Sine EMF condition [*H*(1) = 11.78, *P* < 0.001] (Fig. 2). Conversely, there was no effect on viable cells, nonviable cells, or total cells when utilizing the HEK293 cell line [*F*(2, 46) = 0.265, *P* = 0.768] (Fig. 3).

### BAY K calcium channel activator interacts with Ca^2+^ EMF

**Fig. 4:**
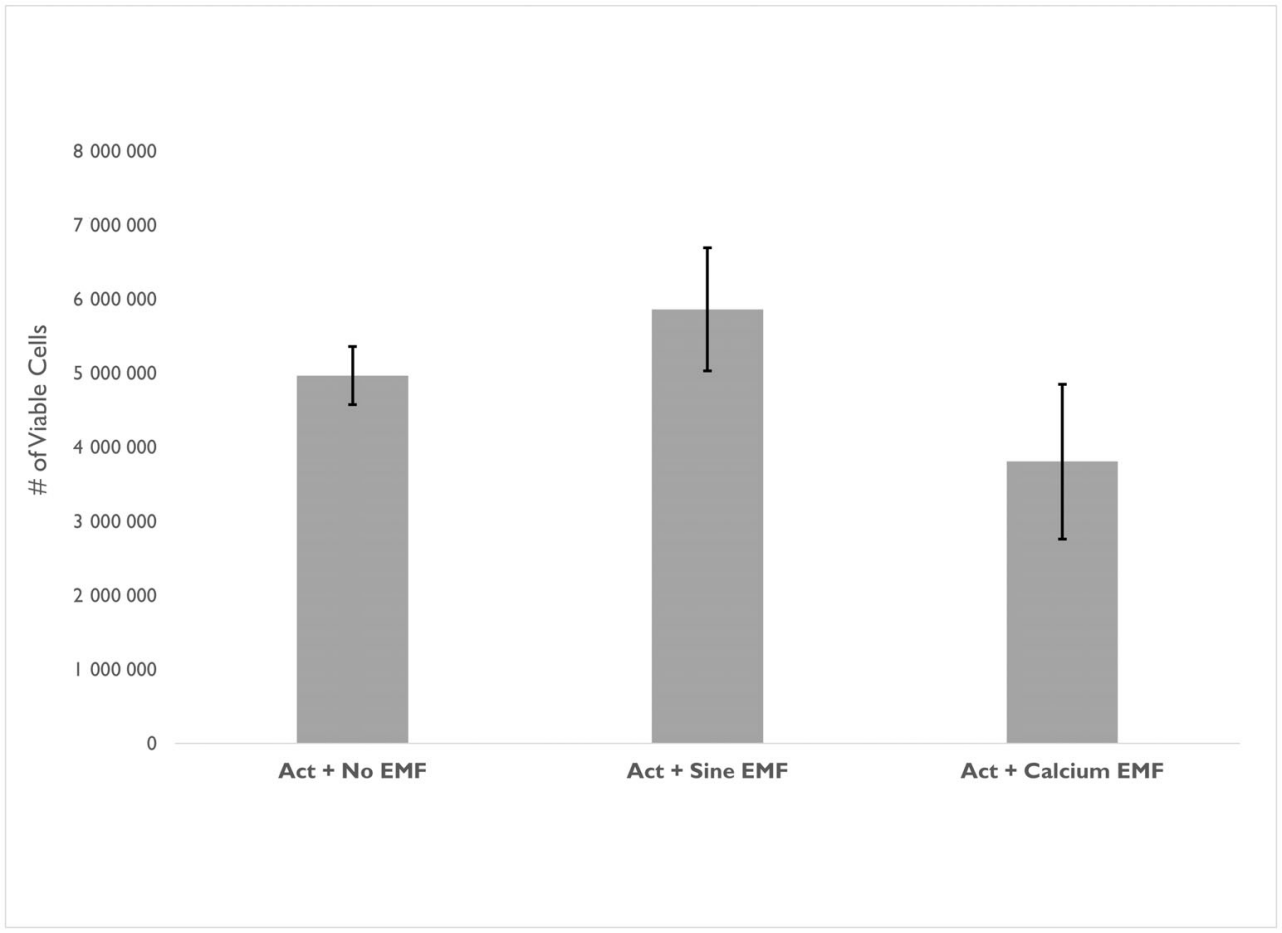
Mean number of viable B16‐BL6 cells by electromagnetic field (EMF) condition with activator. Kruskal–Wallis test: [*H*(2) = 2.838, *P* = 0.242]. Act + No EMF = 6, Act + Sine EMF *n* = 4, Act + Calcium EMF *n* = 6. Error bars represent SEM.

To determine the effect of BAY K8644 on the Ca^2+^ EMF, the data were separated according to the presence or absence of activator (Fig. 4). In the B16‐BL6 cells that received no calcium activator, there was a significant difference between the Ca^2+^ EMF and all other treatment conditions (refer to Section Reduction of B16‐BL6 viability following exposure to Ca^2+^ EMF). In the cells that received the calcium activator and Ca^2+^ EMF, there was no significant difference in the number of viable cells [*H*(2) = 2.838, *P* = 0.242].

### Effect on percentage viability

**Fig. 5:**
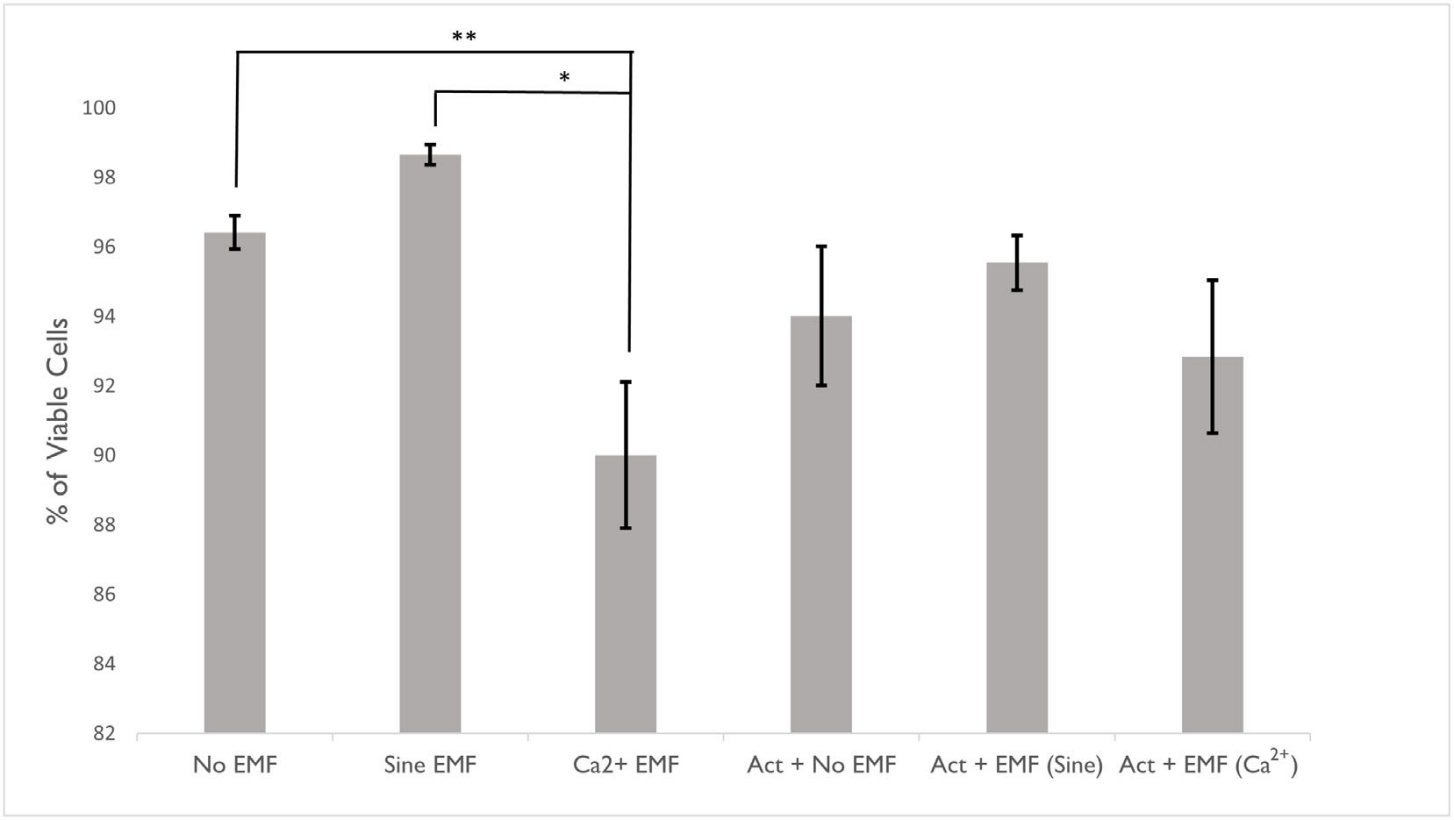
Percentage B16‐BL6 cell viability by electromagnetic field (EMF) treatment condition. Kruskal–Wallis test: [*H*(1) = 9.06, *P = 0.003] [*H*(1) = 11.02, **P < 0.001]. No EMF *n* = 16, Sine EMF *n* = 8, Ca^2+^ EMF *n* = 10, Act + No EMF *n* = 6, Act + EMF Sine *n* = 4, Act + EMF (Ca^2+^) *n* = 6. Error bars represent SEM.

To determine the influence of each treatment condition on the percentage viability (Fig. 5), raw viability scores were used to calculate the percentage of viable cells using the formula provided in the Sigma‐Aldrich hemocytometer protocol [23]. B16‐BL6 cells exposed to the Ca^2+^ EMF condition displayed significantly fewer viable cells when compared to the No EMF condition [*H*(1) = 9.06, *P* = 0.003] and the Sine EMF condition [*H*(1) = 11.02, *P* < 0.001].

### Cell imaging

**Fig. 6:**
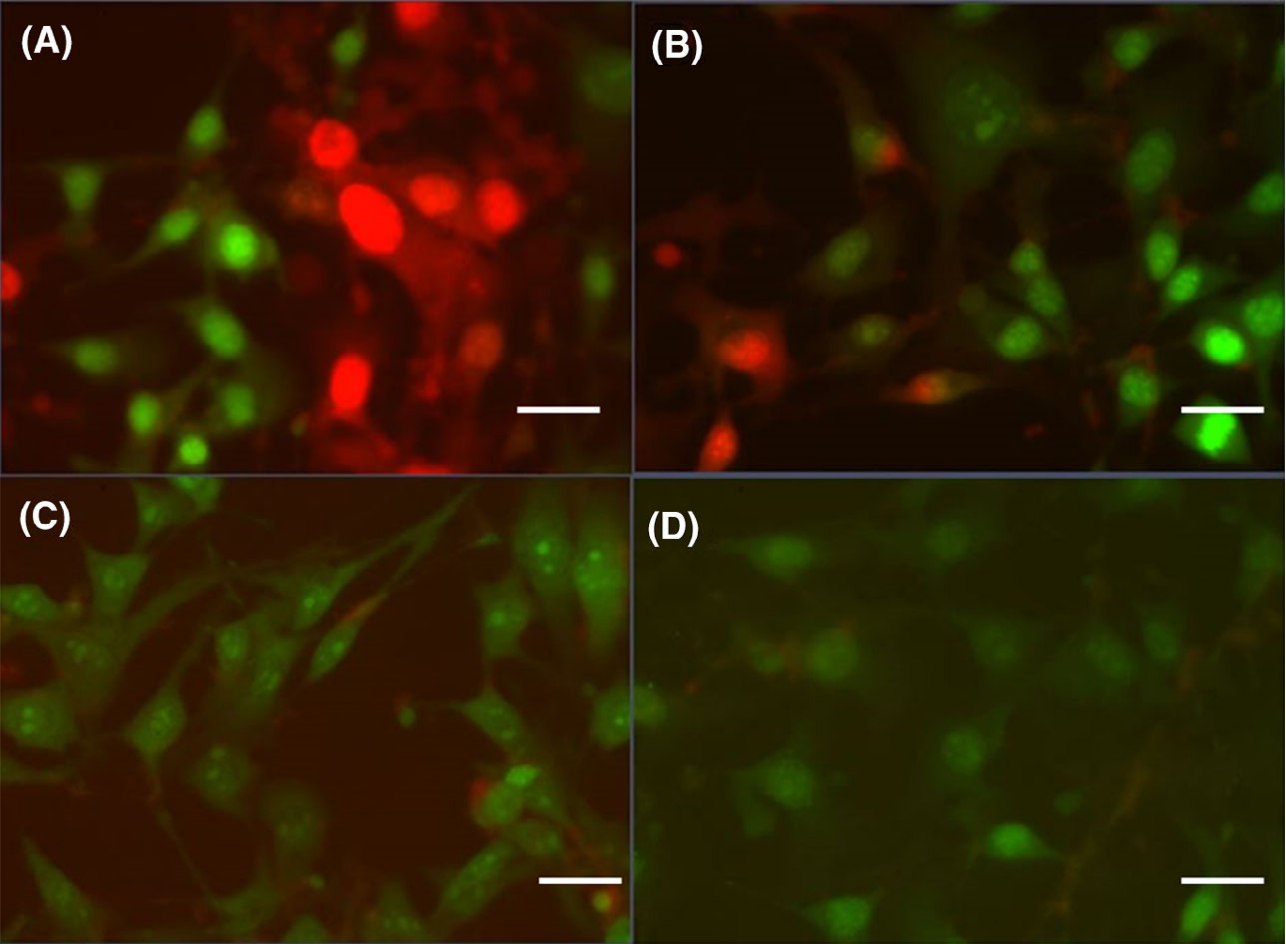
B16‐BL6 cell imaging with acridine orange and ethidium bromide staining. (A) BAY k 8644 + Ca^2+^ EMF, (B) Ca^2+^ EMF, (C) BAY k 8644 + No EMF, (D) No EMF. Scale bar length = 10 μm.

**Fig. 7:**
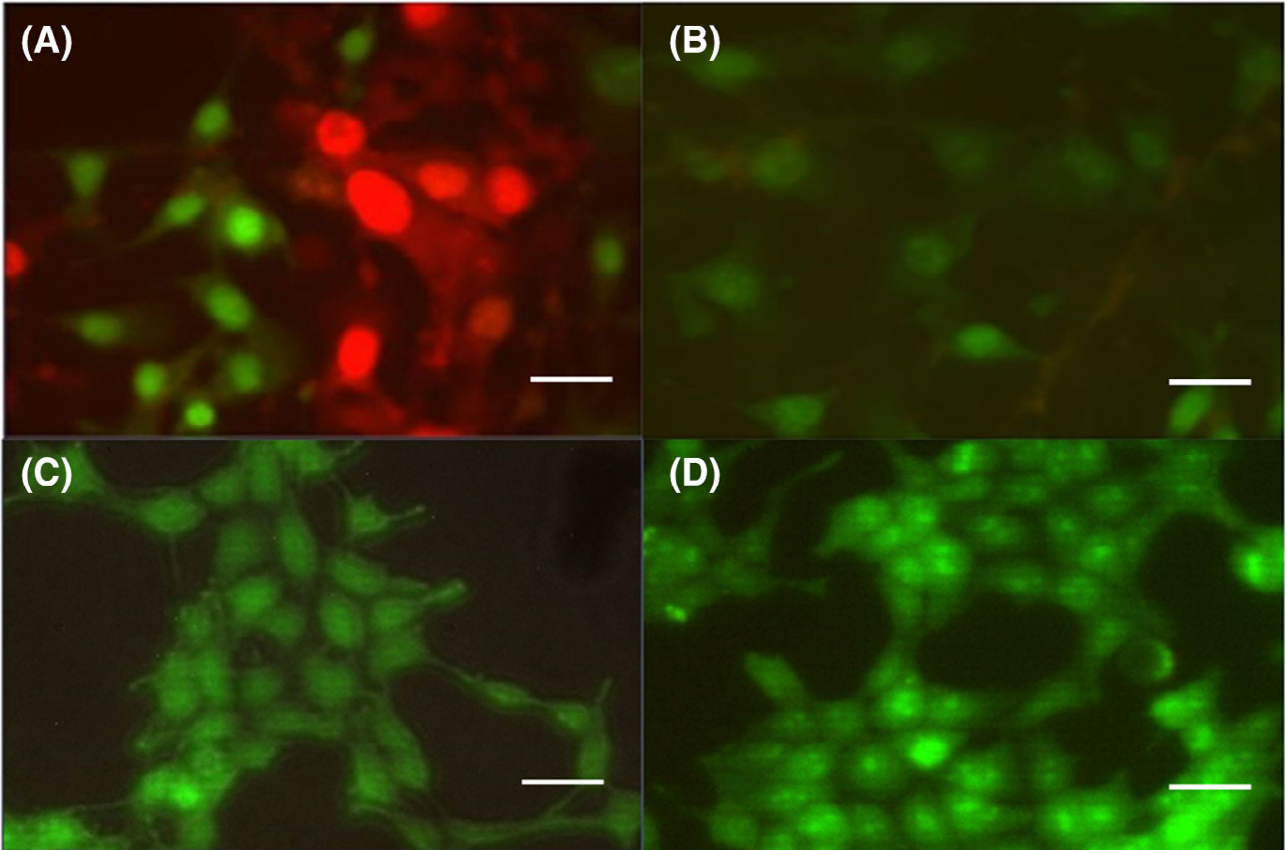
Cell imaging of electromagnetic field (EMF)‐treated B16‐Bl6 and HEK293T cells stained with acridine orange and ethidium bromide: (A) B16‐BL6 + Ca^2+^ EMF, (B) B16‐BL6 cells + No EMF (C) HEK293T + Ca^2^ EMF, (D) HEK293T + No EMF. Scale bar length = 10 μm.

Cell imaging of B16‐BL6 cells subjected to the Ca^2+^ EMF revealed substantial nuclear orange‐red fluorescence by ethidium bromide (EB), while other cells displayed a strong yellow‐green fluorescence in their nuclei by acridine orange (AO) staining (Fig. 6). Additionally, cells under the Ca^2+^ EMF condition appear more uniformly distributed with less aggregation and greater spacing between cells. Similar results were observed by cells in the BAY k 8644 + Ca^2+^ EMF condition, which displayed a large amount of orange‐red, and yellow‐green fluorescence in the cells' nuclei; however, the distribution of cells appeared more variable with cells more tightly packed, forming dense clusters. The intensity of the yellow‐green and orange‐red (AO/EB) stains was greater in the BAY k 8644 + Ca^2+^ EMF condition when compared to all other conditions (Fig. 7).

## Discussion

This is the first known experiment to employ a complex EMF modeled after the physiological firing of ICWs. The results demonstrate that a dynamic, physiologically patterned EMF can inhibit the proliferation of malignant cells and induce cell death. A single exposure of the Ca^2+^‐modeled EMF resulted in a significant decrease in the mean number of viable cells by *50.3%*. The proposed mechanism of action for these specific‐frequency EMFs involves enhancing the presence of reactive oxygen species, differential activation of cellular signaling cascades, and the induction of a rapid Ca^2+^ influx, all of which are proposed to decrease cell growth and induce apoptosis [24, 25, 26]. Furthermore, work by Stratton *et al*. [27], demonstrates that malignant cells exposed to extremely low‐frequency EMFs with an energy of 0.3 μT at a consistent frequency of 10 Hz show a compromised plasma membranes, allowing for an influx of Ca^2+^ that subsequently enhances apoptosis.

The Ca^2+^ EMF is a complex time‐varying pattern housing multiple frequencies that are fluctuating as the field is emitted. As demonstrated in the results, cells exposed to the simple 3 Hz sine wave showed no alteration in proliferation or cell death. Given these findings, it is proposed that the timing and pattern of the frequencies emitted from the EMF to the cells play a crucial role in the field's efficacy to alter proliferation and induce cell death. Conversely, cells treated with the BAY K8644 activator and exposed to the Ca^2+^ EMF did not display a significant decrease in their mean number of viable cells compared with the No EMF group and Bay k8644 + No EMF group. The BAY K8644 calcium activator is a voltage‐gated L‐type calcium channel agonist that increases Ca^2+^ channel permeability, resulting in a brief Ca^2+^ influx [28]. The kinetics of this activator are different from the traditional, long‐lasting action of L‐type calcium channels. The addition of BAY K8644 promotes maximal Ca^2+^ uptake within 10 min of administration, leading to a large increase in cytoplasmic Ca^2+^ concentration, which then declines to resting levels in the following 30 min [6, 28, 29]. The ability of BAY K8644 to inhibit the effects of Ca^2+^ EMF suggests that the efficacy of the field lies in its ability to alter the kinetic properties of L‐type and T‐type voltage‐gated Ca^2+^ channels. Additionally, the lack of an effect with the HEK293 cells is significant. It demonstrates the specificity of the Ca^2+^ EMF mechanism. Cancer is a direct result of alterations in the mechanisms attributed to the proliferation and death of a cell [30]. Since Ca^2+^ signaling is crucial to both processes, the importance of Ca^2+^ kinetics within and between cells cannot be overemphasized [30, 31]. Intracellular increases in Ca^2+^ are central to a variety of cellular signaling events such as Ca^2+^ sparks, oscillations, and waves [12, 32, 33]. The dysregulation of calcium signaling is detrimental to normal cell functioning and is associated with the hallmarks of cancer [34]. The abnormal Ca^2+^ signaling found in malignant cells occurs through the modification and differential expression of Ca^2+^ channels and pumps [15, 33]. While the cellular machinery (Ca^2+^ channels, pumps, and exchangers) is the same in both nonmalignant and malignant cells, the latter may express cellular machinery with alternative isoforms, location, activity, and gene mutations associated with cancerous processes [15]. Modification of this machinery alters the intra‐ and intercellular flux of Ca^2+^ for a given cell as described by the abnormal movement of ions across the plasma membrane [15, 17].

Considering the original Buckner studies [6] and the results from the current study, it is proposed that the mechanism of the Ca^2+^ EMF acts specifically on T‐type VGCCs. Cells exposed to the Ca^2+^ EMF for a 40‐min duration in the absence of the BAY K8644 activator displayed the full anticancer effects. However, cells treated with the BAY K8644 activator prior to field exposure did not demonstrate these properties. In accordance with the findings of Buckner *et al*. [6], we hypothesize the opposing action of the BAY K8644 activator resides in its ability to stimulate a flux of Ca^2+^ through L‐type VGCCs that is necessary for malignant cell survival. Due to the dysregulation in Ca^2+^ signaling and alteration in cellular machinery associated with cancer, the BAY K8644 activator may function to enhance typical malignant Ca^2+^ signaling through altered cellular machinery.

Cell imaging using AO/EB staining revealed a substantial amount of orange‐red and yellow‐green fluorescence in the nuclei of Ca^2+^ EMF‐treated cells (Fig. 6B). As demonstrated by Liu *et al*. [35], cells displaying orange‐red fluorescence in their nuclei are regarded as late apoptotic, whereas those displaying yellow‐green fluorescence are considered early apoptotic. It is important to note that complete apoptosis was not observed from these figures; however, the stark color contrast among different experimental conditions is indicative of different apoptotic stages. The high prevalence of late and early apoptotic cells observed in the Ca^2+^ EMF condition is consistent with the findings of Morrone *et al*. [15], whereby inappropriate flow of Ca^2+^ through T‐type calcium channels resulted in antiproliferative effects and apoptosis in glioma cells. Conversely, images of the Activator + Ca^2+^ EMF group displayed comparable results under AO/EB staining (Fig. 6A). A similar number of early and late apoptotic cells were noted, however, with a more intense fluorescence indicative of nuclear condensation. While both groups appeared to have similar degrees of cellular apoptosis, cells in the Ca^2+^ EMF condition displayed a more uniform distribution with less aggregation and greater spacing between cells. These findings are consistent with the results in Fig. 3, demonstrating a decrease in the mean number of viable cells for the group exposed to the Ca^2+^ EMF.

The presence of apoptosis in the absence of an effect for the BAY K8644 + Ca^2+^ EMF group suggests that there is a temporal component underlying the antiproliferative, cell death‐inducing mechanism of the Ca^2+^ EMF. The Ca^2+^ influx through L‐type channels associated with BAY K 8644 occurs within 10 min of administration and returns to resting concentrations within 30 min. Since administration of the activator suppressed the effects of Ca^2+^ EMF, it is proposed that the field functions by increasing T‐type Ca^2+^ channel permeability, allowing for a gradual Ca^2+^ influx over the 40‐min exposure period. The kinetic action of the BAY K 8644 activator causes a transient Ca^2+^ increase through L‐type channels in the same timeframe the field is active. The competing action of the BAY K 8644 activator may be working to promote normal malignant cell Ca^2+^ signaling through altered L‐type channels while Ca^2+^ EMF is working to cause irregular, disruptive signaling through T‐type channels. We propose that these two competing actions lead to substantial interference whereby the residual effect is negligible.

Findings from this study further support the hypothesis that complex‐patterned EMFs can influence cells, their cohorts, and entire biological systems. These data demonstrate the ability of a physiologically patterned EMF modeled after the activation frequency of Ca^2+^ waves to seize proliferation and induce apoptosis in a malignant cell model. We propose that exposure to Ca^2+^ EMF alters T‐type Ca^2+^ channel permeability, allowing for an irregular Ca^2+^ influx capable of disturbing proliferation and inducing apoptosis of malignant cells. Future studies should investigate the specific mechanisms and timing at which the Ca^2+^ EMF alters calcium channel permeability. Such information would provide valuable insights into our current observations, suggesting that the Ca^2+^ EMF could act as a potential anticancer therapy.

## Conflict of interest

The authors declare no conflict of interest.

### Peer review

The peer review history for this article is available at https://www.webofscience.com/api/gateway/wos/peer‐review/10.1002/2211‐5463.13760.

## Author contributions

BDR and BTD contributed to the conceptualization, formal analysis, and writing—review and editing. BDR, RML, and BTD contributed to the methodology. BDR, ADP‐K, and BTD contributed to the investigation. RML and BTD contributed to the resources and project administration. BDR contributed to the data curation and writing—original draft preparation. BTD contributed to the supervision.

## Data Availability

The data that support the findings of this study are available on request from the corresponding author. The data are not publicly available due to privacy or ethical restrictions.
